# Antioxidant capacity as a novel biomarker of delirium after cardiac surgery

**DOI:** 10.1192/j.eurpsy.2021.675

**Published:** 2021-08-13

**Authors:** J. Kaźmierski, A. Pawlak, P. Miler, H. Jerczyńska, J. Woźniak, E. Frankowska, K. Woźniak, A. Brzezińska, M. Wilczyński

**Affiliations:** 1 Department Of Old Age Psychiatry And Psychotic Disorders, Medical University of Lodz, Łódź, Poland; 2 Department Of Affective And Psychotic Disorders, Central Clinical Hospital, Łódź, Poland; 3 Department Of Adolescent Psychiatry, Central Clinical Hospital, Łódź, Poland; 4 Corelab Central Laboratory Of Medical University Of Lodz, Medical University of Lodz, Lodz, Poland; 5 Department Of Old Age Psychiatry And Psychotic Disorders, Central Clinical Hospital, Lodz, Poland; 6 Department Of Cardiac Surgery, Central Clinical Hospital, Lodz, Poland; 7 Department Of Cardiac Surgery, Medical University of Lodz, Lodz, Poland

**Keywords:** Cardiac surgery, oxidative stress, Major depressive disorders, delirium

## Abstract

**Introduction:**

Coronary-artery bypass graft (CABG) surgery is known to improve cardiac function and decrease mortality, albeit, this method of treatment is associated with a high risk of postoperative delirium. The pathophysiology of delirium after cardiac surgery is largely unknown.

**Objectives:**

To investigate whether oxidative stress reflected by decreased preoperative and postoperative plasma antioxidant capacity (AC) is independently associated with delirium after cardiac surgery. Furthermore, to assess whether the association between AC and the level of soluble receptor for advanced glycation end-products (sRAGE) exists.

**Methods:**

The patients were examined 1 day preoperatively with the Mini International Neuropsychiatric Interview and MMSE test to screen for depression, anxiety disorders, and for cognitive impairment, respectively. Blood samples for AC and sRAGE levels were collected both preopertively and postoperatively. The CAM ICU and MDAS were used within the first 5 days postoperatively to screen for a diagnosis of delirium.

**Results:**

Postoperative delirium developed in 34% (61 of 177) of participants. Multivariate stepwise logistic regression analysis revealed that patients with low baseline AC are at significantly increased risk of developing delirium. Moreover, preoperative AC levels were inversly correlated with postoperative sRAGE concentrations (Spearman’s Rank Correlation -0.198; p<0.05). The most optimal cutoff values of the preoperative and postoperative AC that predict the development of delirium were 1.720 mM and 1.893 mM, respectively.

**Conclusions:**

Decreased plasma AC levels are associated with delirium after cardiac surgery and inversly correlated with post-surgery sRAGE concentration. This may be an important pathophysiological consideration in the increased risk of postoperative delirium seen in cardiac surgery patients.

